# Irradiation Induced Injury Reduces Energy Metabolism in Small Intestine of Tibet Minipigs

**DOI:** 10.1371/journal.pone.0058970

**Published:** 2013-03-19

**Authors:** Yu-Jue Wang, Wen Liu, Chi Chen, Li-Meng Yan, Jun Song, Kun-Yuan Guo, Gang Wang, Qing-Hong Wu, Wei-Wang Gu

**Affiliations:** 1 Department of Laboratory Animal Center, Southern Medical University, Guangzhou, Guangdong, China; 2 Pearl Laboratory Animal Sci. & Tech. Co. Ltd., Dongguan, China; 3 Department of Hematology, The People’s Hospital of Guizhou Province, Guiyang, Guizhou, China; 4 Department of Ophthalmology, Zhujiang Hospital, Southern Medical University, Guangzhou, Guangdong, China; 5 Department of Hematology, Zhujiang Hospital, The Southern Medical University, Guangzhou, Guangdong, China; 6 Department of Oncology, Affiliated Oncology Hospital of Medical College of Guiyang, Guiyang, Guizhou, China; The University of Tennessee Health Science Center, United States of America

## Abstract

**Background:**

The radiation-induced energy metabolism dysfunction related to injury and radiation doses is largely elusive. The purpose of this study is to investigate the early response of energy metabolism in small intestinal tissue and its correlation with pathologic lesion after total body X-ray irradiation (TBI) in Tibet minipigs.

**Methods and Results:**

30 Tibet minipigs were assigned into 6 groups including 5 experimental groups and one control group with 6 animals each group. The minipigs in these experimental groups were subjected to a TBI of 2, 5, 8, 11, and 14 Gy, respectively. Small intestine tissues were collected at 24 h following X-ray exposure and analyzed by histology and high performance liquid chromatography (HPLC). DNA contents in this tissue were also examined. Irradiation causes pathologic lesions and mitochondrial abnormalities. The Deoxyribonucleic acid (DNA) content-corrected and uncorrected adenosine-triphosphate (ATP) and total adenine nucleotides (TAN) were significantly reduced in a dose-dependent manner by 2–8 Gy exposure, and no further reduction was observed over 8 Gy.

**Conclusion:**

TBI induced injury is highly dependent on the irradiation dosage in small intestine and inversely correlates with the energy metabolism, with its reduction potentially indicating the severity of injury.

## Introduction

Radiation-induced mitochondria damage has been extensively studied in the past decade. Mitochondria are susceptible to ionizing radiation because of radiation-induced reactive oxygen species (ROS) overproduction and mitochondrial deoxyribonucleic acid (mtDNA) damage [1], [2]. Following irradiation exposure, enhanced ROS production leads to mitochondrial dysfunction through the disruption of electron flow and mtDNA, subsequently causes cell apoptosis [3]. However, it is largely unknown whether bioenergetic failure is associated with radiation-induced organ injury. Mitochondria are the major suppliers of ATP to maintain biological function in normal tissue. Therefore, bioenergetic failure induced by mitochondrial dysfunction may play a role in organ injury, including small intestinal tissue.

The small intestine, as one of the most sensitive organs to radiation exposure exhibits various degrees of injury after exposing to different doses of irradiation. Because radiation-induced damage is difficult to be diagnosed and evaluated, the measurement of radiation toxicity in small intestine is still focused on hematopoietic system, such as various types of blood cell count, lymphocyte apoptotic rate, bone marrow biopsy. Intestinal toxicity of radiation has long been the focus in radiation associated diseases. After TBI, documentation of clinical signs and symptoms including the hematopoietic, gastrointestinal, cerebrovascular and cutaneous systems is essential for the therapy and prognosis. Once gastrointestinal syndrome occurs, the patient health becomes detrimental. For abdominal and pelvic tumor patients, the toxicity of radiation in the small intestine becomes the main obstacle for high-dose radiotherapy. Investigation of molecular mechanism underlying the intestinal radiation damage will provide the insight for clinical diagnosis and prognosis.

The purpose of this study is to provide potential parameters to evaluate the irradiation caused organ damage using minipig as the animal modelby providing the experimental evidence to understand the correlation between the energy metabolism and organ injury. Compared with small animal models such as rodents, mini pigs share the high similarity to human in anatomy, development and physiology. Therefore, Tibet minipig was selected as a model system in our studies. To examine the irradiation induced tissue damage and energy metabolism, Tibet minipigs were exposed to TBI with various doses, the adenosine-triphosphate (ATP), adenosine-diphosphate (ADP), adenosine-monophosphate (AMP) and total adenine nucleotides (TAN) were determined at 24 h following exposure. By analyzing energy metabolism and tissue injury caused by TBI, we conclude that energy metabolism correlates with the extent of injury in the small intestine.

## Results

### Irradiation Causes the Reduction of ATP, ADP, AMP and TAN

**Figure 1:**
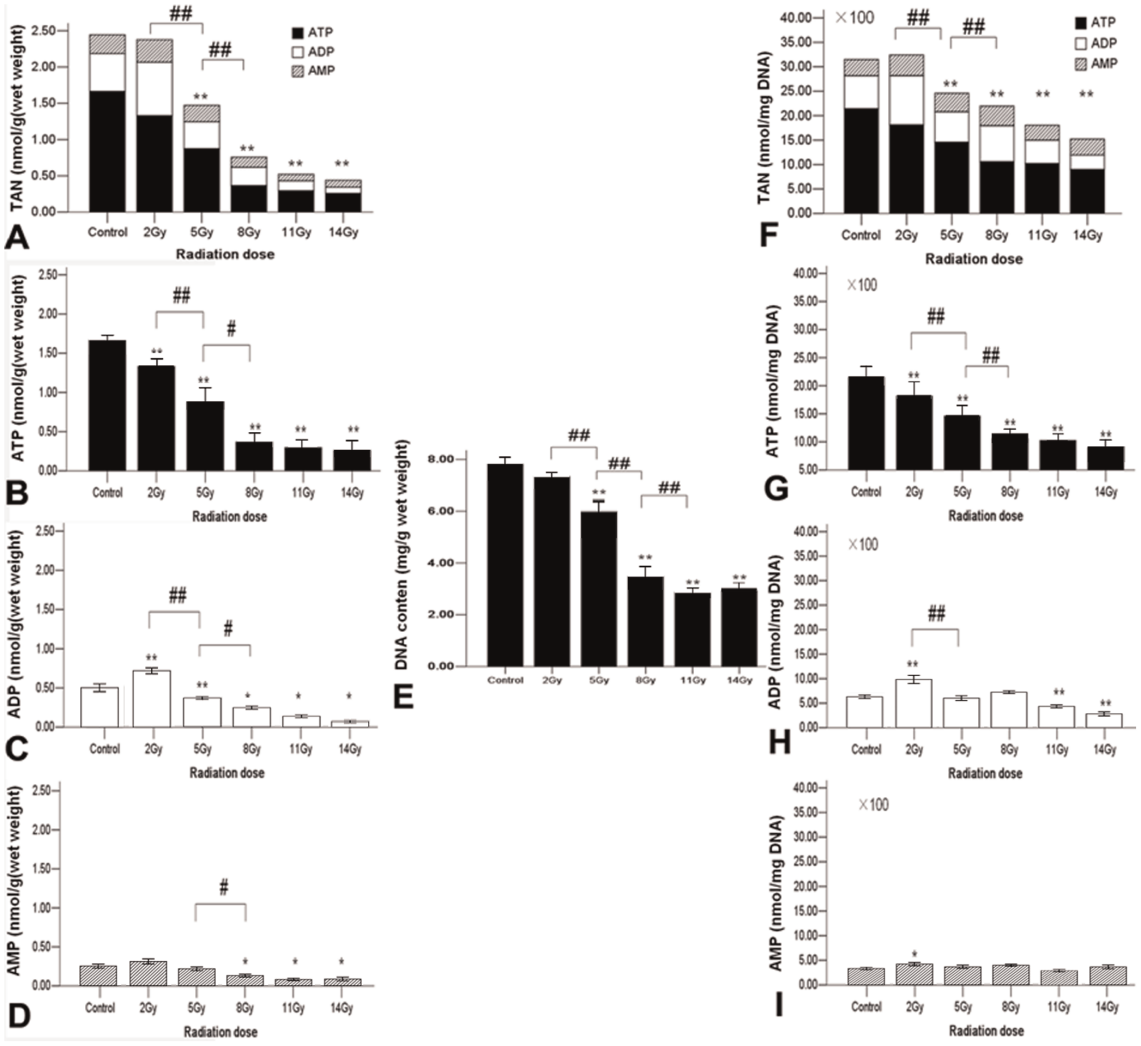
The levels of ATP, ADP, AMP and TAN for different dose irradiated groups and control group at 24 **h post-TBI.** The bars represent the means of energy substances. X axis indicates the content of energy substances and Y axis indicates the different group. *: p<0.05, **: p<0.01(versus control); ^#^: p<0.05, ^##^: p<0.01 (post hoc LSD or Games-Howell test).

Radiation dose had significant effects on ATP (nmol/g wet weight), ADP (nmol/g wet weight), AMP (nmol/g wet weight) and TAN (nmol/g wet weight) at 24 h post-TBI (all p<0.01) (Fig. 1A–D). ATP contents in all irradiated groups were lower than in the control group. ADP content in the 2 Gy group was higher than in the control, whereas it was lower than the control in 5, 8, 11 and 14 Gy groups. AMP contents were significantly reduced in 8, 11 and 14 Gy, whereas no significant differences were found in 2 and 5 Gy groups compared with control. TAN contents in all irradiated groups were lower than that in the control group except 2 Gy group (control vs 2 Gy p>0.05). In addition, the contents of ATP, ADP and TAN displayed radiation dose-dependent reduction for the doses of 2–8 Gy and no significant difference for the doses of 8–14 Gy.

### DNA Contents in Small Intestine are Significantly Reduced Following Irradiation

Different radiation doses had significantly different effects on small-intestinal DNA content at 24 h post-TBI (p<0.05) (Fig. 1 E). DNA contents in all irradiated groups were lower than in control except for the 2 Gy group (2 Gy vs control p>0.05). A radiation dose-dependent decrease in DNA content was observed for the doses of 2–11 Gy. However, there was no significant difference in DNA content between 11 and 14 Gy (11 Gy vs 14 Gy p>0.05).

### Irradiation Causes the Reduction of ATP, ADP, AMP and TAN Corrected by DNA Contents

To further verify whether the reduction of ATP, ADP or AMP content actually reflects the reduction of energy production or solely the radiation-induced reduction of the cellular component in the irradiated intestine, the ATP, ADP, AMP and TAN were corrected by small-intestinal DNA content (Fig. 1, F–I). Following the correction, ATP (nmol/mg DNA) in all irradiated groups was lower than that in the control group and exhibited a radiation dose-dependent decrease from 2–8 Gy, whereas no significant differences were found in the doses of 8–14 Gy. ADP (nmol/mg DNA) was higher than the control for the 2 Gy group and lower than the control for 11 and 14 Gy, while no significant differences were found for 5 and 8 Gy (5 and 8 Gy vs control, p>0.05). AMP (nmol/mg DNA) was higher in the 2 Gy group than that in the control and no significant differences were found for 5, 8, 11 and 14 Gy. TAN (nmol/mg DNA) was found to have the similar trend as for the uncorrected TAN results.

### Histological Study

**Figure 2:**
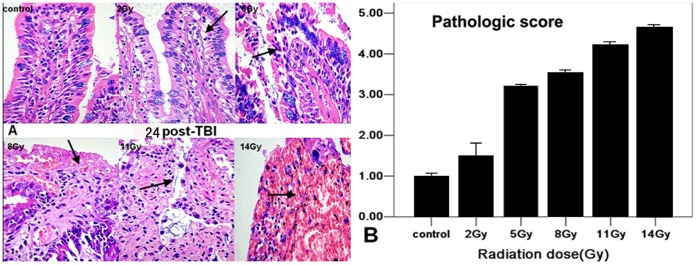
Histological analysis and pathological scores in small intestine. **A.** the pathologic alterations in the various doses of irradiated groups and the control group at 24 h post-TBI were observed in H.E. stained sections of small intestine.(arrows indicate apoptosis and hemorrhage) e (×400). **B.** pathologic alterations in the sections of small intestine were scored and graphed. Y-axis indicates pathologic severity.

**Figure 3:**
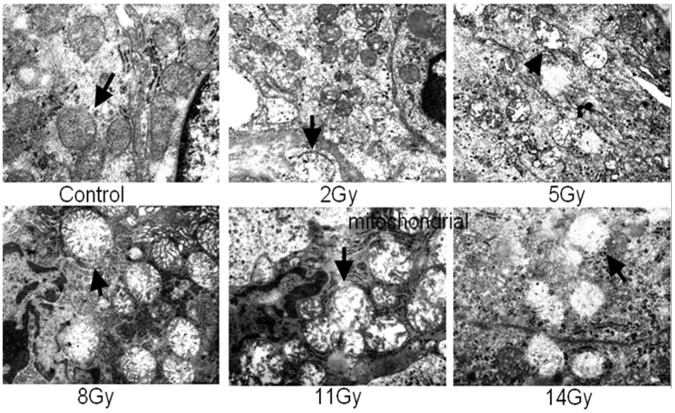
Ultrastructure of mitochondria. Compared with control group, electron micrographs show enlarged mitochondria and reduced electron density for 2 and 5 Gy. The increased electron lucent and vacuolization were shown for 8, 11 and 14 Gy. (×15 000–20 000).

Microscopic examination (Fig. 2A) of tissue from 2 and 5 Gy groups revealed that compared with control, the lumen structures of small intestine were slightly damaged, edema and dilated hyperanemia localized in the lamina propria and submucosa of small blood vessels. The small intestinal villi were sparse and necrosis was found in the crypt epithelial cell. Moreover, the cytoplasms of lamina propria cells were eosinophilic. In contrast to the 2 and 5 Gy groups, lesions were more severe in the 8, 11 and 14 Gy groups with severely damaged mucosal structures, thinner intestinal wall, epithelial necrosis, congestion and mucosal or submucosal bleeding. From the results of semi-quantitative pathologic assessment, the scores were increased and correlated with radiation doses (Fig. 2B). The overall ultrastructure of the small intestine was well-maintained without autolytic artifacts (Fig. 3). For 2 and 5 Gy irradiated groups, cristae had lost contact with the outer mitochondrial membrane and the aberrant mitochondria were three to eight times larger than the normal size. For 8, 11 and 14 Gy groups, the obvious electron lucent and vacuolization were observed.

### Correlation Analysis of Energy Productions and Pathologic Score at 24 h Post-TBI

**Figure 4:**
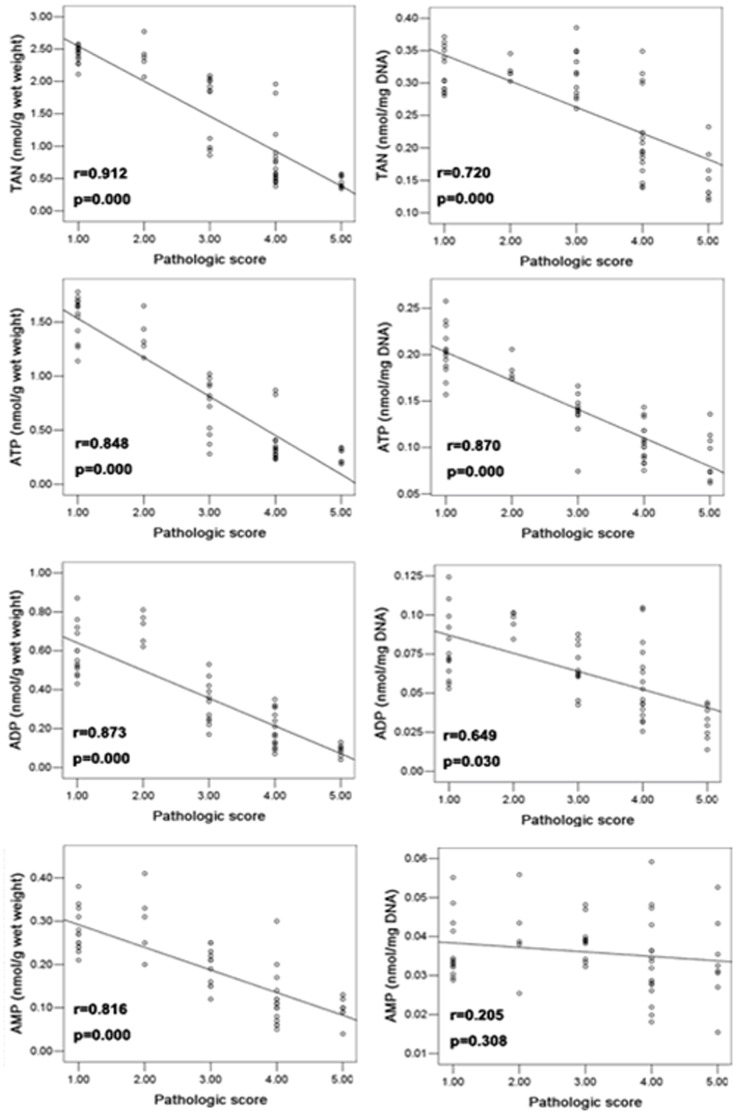
Correlations of pathologic scores (Ph score) and concentrations of energy substances at 24 h post-TBI. All of uncorrected energy substances were negatively correlated with pathologic scores (Spearman test) at 24 h post-TBI. All of DNA content-corrected energy substances were negatively correlated with pathologic scores (Spearman test) at 24 h post-TBI except DNA content-corrected AMP.

The correlation of energy substances with pathologic scores was analyzed by Pearson correlation analysis (Fig. 4). The negative correlations between all of energy productions (including TAN) and pathologic score were observed in DNA-corrected and non-corrected ATP, ADP and TAN.

## Discussion

### Small-intestinal Energy Metabolism after TBI

Irradiation induced cell death is involving in gene activation, protein modification, ion concentration changes and many other factors. It is known that the radiation-induces cell death due to cellular DNA damage, which activates p53 gene expression and promotes apoptosis in thymocytes [5]. DNA damage can also stimulate calcium influx, thereby activate the endonuclease to induce DNA degradation and programmed cell death [6]. Radiation damage enhances membrane permeability, alters cell shape, causes the swelling of endoplasmic reticulum and mitochondria, and thereby leads to lysosomal destruction. Our experiments showed that the radiation induced DNA damage was highly dose dependent and associated with the cell and organ damage [7].

While previous studies have demonstrated that radiation exposure could cause small-intestinal injury by inducing apoptosis, some reports support a role of radiation-induced bioenergetic failure in the process of injury. In this study, for the first time, we observed the radiation dose-dependent bioenergetic failure and the negative correlation between energy productions and pathologic findings. By further analysis of these findings, our results suggest that the major reduction of energy substances affected ATP. The transient rise of ADP and AMP at 2 Gy dose may be caused by the transformation of ATP into ADP and AMP to produce more high-energy phosphate bonds in order to meet the greater energy demand under external stress in the small intestine. It is well-known that the mechanism of radiation-induced toxicity in the GI tract is the death of GI epithelial cells [4]. In addition, several studies revealed that mitochondria are major players in the regulation of cellular processes, including oxidant-mediated apoptotic signaling [2], [3], [8]. Therefore, the marked reductions of mitochondria and energy production (particularly ATP) for 8, 11 and 14 Gy may contribute to the life-threatening consequences and poor treatment in response to TBI induced injury in the small intestine. Previous studies indicated that exposure of the body to 6–8 Gy irradiation significantly reduced leukocytes and platelets, resulting in hemorrhage and death within 30 days of exposure. Morbidity was significantly increased when the body was exposed to a high dose (≥10.4 Gy) in a short period of time, and the victims suffered irreversible hematopoietic and gastrointestinal injury. The threshold of irradiation is of particular significance, since clinically at radiation doses of 3 to 8 Gy, the victims can be rescued by bone marrow transplantation and cytokine administration [9]. In contrast, a whole body exposure of ≥10.4 Gy induces 100% mortality within 10–15 days without medical countermeasures for rescue. Given that the radiation-induced energy metabolism failure contributed to intestinal injury, our findings suggest that the improvement or/and inhibition of small-intestinal energy metabolism may be an approach to rescue the victims with high dose irradiation exposure.

### Correlation between Energy Production, Pathologic Lesion, and the Potential Application

In our previous study, we found TBI caused a systemic organ reaction, different organs respond differently. The main character of immune system such as spleen and lymph node showed signs of shrinkage and apparent peripheral hemorrhage with the increased radiation doses. The lymphocytes in these organ showed nuclear condensation, fragmentation and dissolution [10], [11]. In this study, our results showed that acute radiation of the small intestine caused mucosal lesion and hemorrhaging in a dose-dependent manner, which is in agreement with a previous study using rodents [12]. By comparing the content of energy production and the lesion severity of small intestine following TBI, we found that the DNA content-corrected ATP and TAN were significantly correlated with lesion severity. In addition, the uncorrected ATP, ADP, AMP and TAN were correlated with the lesion severity as well. Therefore, the correlation between small-intestinal energy metabolism and pathologic lesion may provide a potential parameter to assess the intestinal damage and viability. Phosphorus-31 magnetic resonance spectroscopy (^31^P-MRS), as a noninvasive and nondestructive approach, was used to observe the change of tissue ATP clinically and in animal models [13]–[16]. Our future study will examine whether ^31^P-MRS could be used for early assessment and monitoring of the radiation-induced response in the small intestine.

In conclusion, both the DNA content-corrected and uncorrected ATP levels showed a negative correlation with pathologic lesion. Early radiation response of energy metabolism can be potentially used in humans for early assessment of absorbed radiation doses and pathologic lesion in radiation-induced small-intestinal damage.

## Materials and Methods

### Animals and Ethics Statement

This study was carried out in accordance with the Guidelines for the Care and Use of Laboratory Animals of the National Institutes of Health. A total of 30 adult male (8 to 15 month-old) Tibet mini pigs were purchased from the Laboratory Animal Center of Southern Medical University of China and used for TBI exposure (The protocol was approved by the Committee on the Ethics of Animal Experiments of the Southern Medical University of China (Protocol Number: 2010040). All surgery was performed under ketamine anesthesia.

### Radiation Protocol

All pigs were divided into six groups randomly and anesthetized by using ketamine (0.05 ml/kg i.v.) before radiation exposure. One control group (n  = 6) were not exposed to X-ray. Five treatment groups (n = 6 for each group) were irradiated with single dose of 2, 5, 8, 11 and 14-Gy at the dose rate of 255 cGy/min, respectively using a 8-Mv X-ray linear accelerator (Elekta Synergy Platform, ELEKTA Ltd, Sweden). The linear accelerator has been standardized by CRS-3D tank system (MED-TEC USA) before radiation. Radiation field was calculated as 2ab/(a+b) (a: length, b: width). Source-axis distance was 100 cm; the source-surfaced distance (SSD) was 85 cm (thickness of mini pigs was presumed to be 30 cm). The conversion formula of given dose and the machine output was the following: Dm = DT/TPR×SADF×Sc.P (TPR: tissue phantom ratio;SADF: source-axis distance factor; Sc,P: total scatter calibrate factor; Dm: monitor unity (MU) dose; DT: tumor dose). Minipigs were sacrificed at 24 h post-TBI by bleeding and samples were collected for test.

### The Measurement of DNA Content

The mucous membrane of the small intestine was excised and weighed after removing fat and connective tissues and then washed with ice-cold phosphate-buffered saline (PBS). All of these samples were collected in the same part of small intestine. DNA was measured according to methods described in previous study [18]. Calf thymus DNA was used as a standard.

### Energy Substance Detection

Chemicals including Perchloric acid (HCLO_4_), caustic soda (NaOH), potassium dihydrogen phosphate (KDP), methanol, acetonitrile, tetrahydrofuran were obtained from Guanghua Co, Ltd (Shanghai, China). Standard substance of ATP, ADP and AMP was obtained from Jince Analytical Technique co. Ltd (Tianjin, China).Water used for the buffer preparation was obtained from a Milli-Q water system (Millipore, Bedford, MA, USA). All rinsing solutions were filtered before use through 0.45 mm nitrocellulose Millipore filters. All solutions were prepared and used on the same day.

### High Performance Liquid Chromatography (HPLC) Apparatus

The overall analytic system consisted of a type-LC-10AT HPLC apparatus (Shimadzu, Japan), a K-2501 Spectra System photodiode array UV detector (KNAUER, German), a type-HW-2000 Spectrum Work Station 2.17 (Qianpu, Nanjing, China), a type-828 ORION MODEL Ph detector (USA).

### Preparation of Small Intestine Samples

The samples of small intestinal tissue were placed in liquid nitrogen to prevent any enzymatic degradation (about 20 min). The isotonic Na chloride (1.0 ml) was mixed with the small intestine (100 mg) and then 0.5 Mol/L HClO_4_ (0.1 ml) was added for homogenization on ice. The homogenized mixture was centrifuged for 15 min (8000 r/min). The supernatant was transferred into another Eppendorff tube, and then 1 Mol/L NaOH was added to adjust the PH value to 7. The supernatant was centrifuged for 10 min (8000 r/min) again, and kept at −20°C for testing.

### HPLC Standards

HPLC standards were as follows: 1) Chromatographic column: SHIMADZU C30 column (4.6 mm×250 mm, 5 µm), 2) Mobile Phase:phosphate buffer (PB) (KDP 1.36 g+0.1 Mol/L NaOH 15.2 ml, were diluted by distilled water to 100 ml), flow speed was 0.9 ml/min, the mobile phase was injected in helium at the speed of 15 ml/min for continuous degassing, 3) Wavelength was 254 nm, 4) sample volume: 20 µL,5) Column temperature: 20°C, 6) Testing sensitivity: 1 UAFS.

### System Suitability Test

A certain amount of standard substances of ATP, ADP and AMP were each weighed and diluted by PB (Mobile Phase) to 25 mg/L liquor. 20 µl of the liquor was added to the HPLC test according to HPLC standards. Peak area was then calculated. The mixture reference solution containing 25 mg/L ATP, ADP and AMP was prepared. Then the 20 µL of mixture reference solution, mixture reference solution plus small intestine supernatant, and small intestine supernatant were tested by HPLC according to the standard described above.

### Preparation of the Standard Curve

Weighed a certain amount of standard substances of ATP, ADP and AMP respectively, and diluted by PB (Mobile Phase) to a series of content mixture reference solution, added 5, 10, 15, 20, 25 µl, respectively. The regression equation and correlation coefficient were calculated by the volume of standard sample and peak area.

### Precision and Stability Test

Tissue collected from the same animal was equally divided into three portions, and each was offered the same sample processing and analysis twice. Then the coefficients of variation (RSD) of the ATP, ADP and AMP values were calculated using data obtained from the six tests.

### Recovery Test

The 6 samples of small intestine were collected from the minipigs randomly, and divided into two groups equally. One group (0.5 ml) was added mixture reference solution for test with the contents of 20, 25, 30 mg/L respectively, another group (0.5 ml) was tested without any treatment. The recovery was calculated by comparing the difference of peak volume between the two groups [17].

### Measurement of ATP, ADP and AMP

ATP, ADP and AMP content per gram of small intestinal tissue (wet weight) were calibrated according to the standard graph, then the TAN value was calculated according to the formula: TAN = ATP+ADP+AMP. In addition, the result of ATP, ADP, AMP and TAN was standardized by DNA content in the intestine to control the interfering factor (radiation-induced apoptosis in mucosal cells of small intestine).

### Histological Study

The samples of small intestine were fixed in 10% PBS buffered formalin solution for 48 h and dehydrated in different grades of ethyl-alcohol and embedded in paraffin. Two-micrometer sections were cut and stained with hematoxylin-eosin. The pathological grading of intestinal lesions was quantified according to previous literature [19]: 1) no significant abnormality was scored 1; 2) mild abnormality (superficial mucosal erosion, inflammatory infiltrate within the lamina propria) was scored as 2; 3) moderate abnormality (mucosal ulceration, intact muscularis propria) was scored as 3; 4) Severe (localized) abnormality (mucosal ulceration, focal hemorrhage) was scored as 4; 5) Severe (generalized) abnormality (mucosal ulceration and diffuse hemorrhage) was scored as 5; The small intestines for transmission electron microscopy (TEM) were fixed in glutaraldehyde solution (2.5%), and then in uranyl acetate (0.5%), followed by dehydration and embedment in resin. The samples were cut into semi-thin and ultra-thin longitudinal sections, and examined by TEM. (JEOL 1010, Jeol, Tokyo, Japan).

### Statistical Analysis

Data are presented as means ± standard deviation (SD) as indicated. One-Way ANOVA test was used for data analyses. Multiple comparisons within each group were measured by least significant difference (LSD) test or Games-Howell test determined by homogeneity of variance test. P values (less than 0.05) is considered statistically significant (two-tailed). Pathological analysis was measured using a nonparametric K independent sample test. The correlations of energy materials with pathologic score were tested by Spearman test. All data were processed using Statistical Product and Service Solutions (SPSS 11.0)software.
